# A Vehicle-to-Grid System for Controlling Parameters of Microgrid System

**DOI:** 10.3390/s23156852

**Published:** 2023-08-01

**Authors:** Jigar Sarda, Yashrajsinh Raj, Arpita Patel, Aasheesh Shukla, Satish Kachhatiya, Mangal Sain

**Affiliations:** 1 M. & V. Patel Department of Electrical Engineering, Chandubhai S. Patel Institute of Technology, Charotar University of Science & Technology, Anand 388421, Gujarat, India; jigarsarda.ee@charusat.ac.in (J.S.); 19ee018@charusat.edu.in (Y.R.); 19ee006@charusat.edu.in (S.K.); 2 V. T. Patel Department of Electronics & Communication Engineering, Chandubhai S. Patel Institute of Technology, Charotar University of Science & Technology, Anand 388421, Gujarat, India; arpitapatel.ec@charusat.ac.in; 3 Department of Electronics and Communication Engineering, GLA University, Mathura 281406, Uttar Pradesh, India; aasheesh.shukla@gla.ac.in; 4 Division of Computer & Information Engineering, Dongseo University, Jurye-ro, Sasang-gu, Busan 47011, Republic of Korea

**Keywords:** electric vehicle (EV), renewable energy sources (RESs), microgrid, vehicle to grid (V2G), photovoltaic (PV), wind

## Abstract

The power system for large-scale adoption of hybrid electric vehicles can benefit from a distributed reserve provided by the vehicle-to-grid (V2G) concept. This study suggests a V2G technology that can effectively control frequency on a microgrid throughout a 24-h cycle. When usage is at its lowest in the spring or fall, a microgrid is intended to be large enough to simulate a community of 2000 households. A 1:5 ratio of cars to households is realized by modelling 400 electric vehicles (EVs) as a basic model, indicating a typical case in the future. An in-depth analysis of the voltage, current, reactive, and active power is carried out for a microgrid. By coordinating control of diesel generation, renewable energy source (RES) generation, power exchange, and EV generation, the system frequency of a microgrid can be managed by regulating load demand with V2G devices. The proposed microgrid with V2G effectively manages energy and reduces the uncertain and variable nature of RES power generation with enhanced performance. System parameter variations have been investigated for various operating scenarios, and it has been discovered that error is confined to less than 5%.

## 1. Introduction

**Figure 1:**
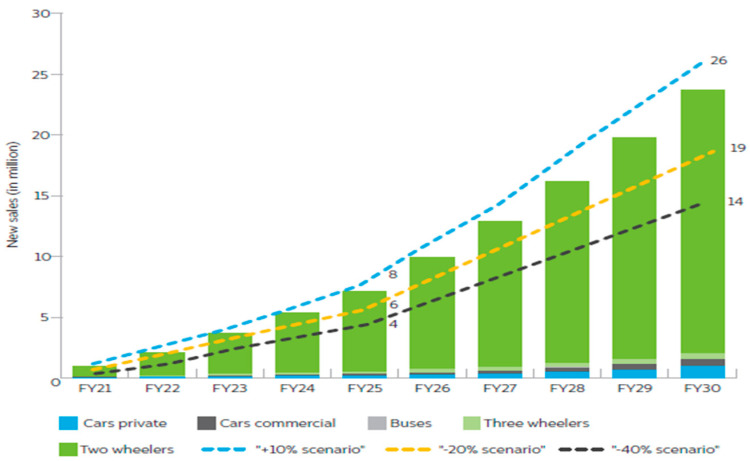
EV sales grow under different scenarios.

One of the significant issues in the energy sector in the most developed nations is the decarbonization of transportation fleets [1,2,3,4]. There is a strong trend toward integrating RESs with EVs and considering V2G in the smart grid as a solution to reduce dependency on petroleum products and meet energy demand. The NITI Aayog’s 2030 electric transportation strategy for India [5] represents a substantial market opportunity. The report analyzes EV sales by market segment, battery needs, required public charging infrastructure, and investments required through 2030 to facilitate India’s EV transition. The study simulates three alternative transition scenarios in addition to the 2030 goal. If this ambition is realized, India’s EV industry might play a significant role in the country’s post-COVID-19 economic recovery. Along the entire value chain, including in the existing and new industries, it can create jobs and economic value. India’s 2030 e-mobility vision, which calls for 70% of all commercial vehicles, 30% of private vehicles, 40% of buses, and 80% of two- and three-wheeler sales to be electric, amounts to 26 million EVs. The EVs are anticipated to make up 43% of all new car sales under the high adoption scenario, 10% over the objective up to 112 million units. In a scenario with limited adoption, this may drop to 23% for new car sales, 40% below the desired level, up to 61 million units. Figure 1 shows the growth of EV sales under different scenarios.

The adoption of an electric fleet of vehicles offers various benefits for the environment and the economy, but widespread EV adoption could cause significant power system fluctuations, especially during times of high demand [6]. Only 5% of EVs are typically driven daily, while the other 90% are idle and stored in lots [7]. To encourage the active participation of EV owners in revenue generation, different countries have provided different kinds of incentives, shown in Table 1.

**Figure 2:**
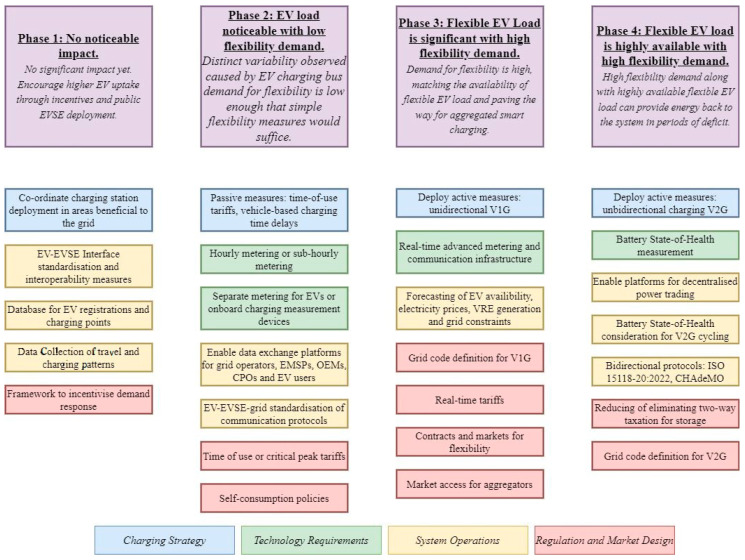
Framework for grid integration of EVs [14].

The number of fossil-fuel products used in conventional power plants to produce electricity is reduced in the first place. In the second place, the use of internal combustion (IC) automobiles moves to EVs, reducing the amount of gasoline. Numerous studies have used simulation modules, particularly the Monte-Carlo Simulation [9], while others have created novel optimization models to assess the current power network’s suitability for adopting a fleet of electrified vehicles in the future [10,11]. Reducing peak demand and boosting grid reliability are two of the V2G system’s most significant goals [12,13]. International Energy Agency has developed a framework for grid integration of electric vehicles to encourage the active participation of EV owners in grid stability, shown in Figure 2.

Drude, Pereira Jr., and Ruther [15] used a MATLAB simulation to examine how the V2G in metropolitan locations affected the energy demand profile in various charge and discharge modes. To create a flatter power demand curve, Khemakhem, Rekik, and Krichen [16] thought of using regulated charge for plug-in electric vehicles (PEVs) as a supervisory method to create a flatter power demand curve. By using electric car aggregators with V2G capabilities, Lopez et al. [17] presented an optimization model for power-load shifting in smart grids. To maximize the performance of EVs and smooth out the sporadic nature of RESs and energy cost reduction, Mehrjerdi and Rakhshani [13] introduced nonlinear stochastic programming. Additionally, this approach reduces the number of times the car batteries are charged and discharged, preventing battery deterioration. Trorja et al. [18] examined how RES penetration, emissions, and balancing power plant performance are affected by energy storage systems and V2G. Hoehne and Chester [19] studied the effects of discharging and charging cycles on emissions and found that optimal EV charging lowered the number of emissions in many cases.

Power networks based on conventional power plants have a consistent power supply since they typically do so throughout the day. The strength of this study, compared to previous research, by providing fresh and cutting-edge indices for monitoring these characteristics, is assessing the effects of the V2G system on the emission, cost, and reliability of the grid for different types of energy sources. However, because RESs are intermittent, power networks that rely on them as their main source of produced energy have an erratic supply pattern. Another significant addition to this study is the stochastic power supply, where two innovative indices are introduced for assessing the system’s dependability in the context of two different power grid types—the grid with less penetration of RESs and the grid with more penetration of RESs. Reliability and environmental and economic elements of this subject are all considered in this study as a thorough analysis is provided on the effects of EVs, the V2G system and its integration with RESs on the electricity grid, particularly wind and solar generating. Many scenarios are specified, and a Monte-Carlo simulation provides them with the results of the system performance measurements. Based on the power grid’s characteristics, the recommended methodology, simulation, and indices may be used in many places and provide excellent tools for policymakers.

There are some potential challenges with the scaling up of EV adoption discussed by [20], which are listed below:

1. Charging Infrastructure: Tie [21] advises constructing a thorough, national charging infrastructure before introducing EVs since it is a critical problem regarding the adoption of EVs.
2. Charging Stations: Several studies have determined that a lack of infrastructure directly impacts customers’ intent to buy an EV [22,23,24]; this affects market sales and ranks at the top of the list of objections against the widespread use of EVs [25].
3. Repair and Maintenance Workshops: Quak, Nesterova and Rooijen [26] claim that existing EV owners are dissatisfied with the lack of EV maintenance facilities and workshops compared to those for ICEVs.
4. Driving Ranges and Charging Times: The restricted capacity and driving range of the batteries as well as their high cost, are two of the critical problems with BEVs. Although some new models have ranges up to 400 km, and subsequent models are projected to have ranges beyond this, the battery size of many existing models restricts their driving range to 250 km [27].

This work is structured as follows: Section 2 discusses the architecture of the V2G interconnected microgrid. The test system is introduced, and results are discussed in Section 3. Finally, Section 4 concludes.

## 2. Architecture of Projected V2G Interconnected Microgrid

A 24-h simulation may be performed quickly using a MATLAB power system network phasor model. A microgrid consists of four sections: the system’s primary power source is a DG, a PV coupled with a wind, which provides RES, and a V2G arrangement, which serves as an additional load in the grid. In the spring or autumn, during the days when energy use is low, the size of a microgrid is to serve a community made up of 2000 households. The default model considers 400 EVs, meaning a 1:5 ratios between the EVs and homes, which would be considered a good future configuration.

**Figure 3:**
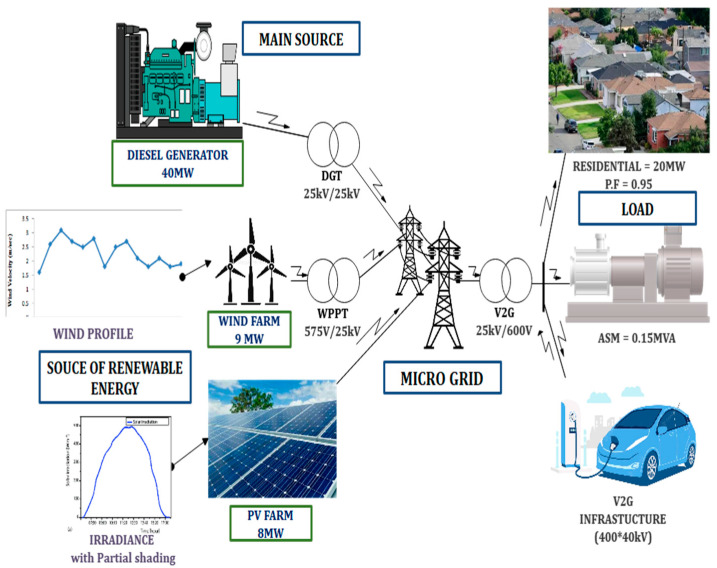
A simple structure of the V2G model.

Figure 3 depicts a schematic representation of the planned microgrid. The electricity provided by RESs, V2G, and consumed power are all balanced out by the diesel generator. Between the grid and the diesel generator, there is a 53 MVA isolation transformer DGT is placed. A 9 MW wind power facility, is linked by a transformer WPPT, which has ratings of 12 MVA and 0.575/25 kV. Between the grid and V2G, there is a 53 MVA transformer is placed. Residential loads with a 20 MW capacity and an Asynchronous Machine (ASM) with a 0.15 MW rating are linked to the distribution side, which runs at 600 V. The battery charge power generating units and grid control are integrated with the V2G, which has a 16 MW rating.

### 2.1. Diesel Generator

The DG helps maintain equilibrium between the amount of energy produced and used. Its synchronous unit’s rotor speed could be utilized to determine the grid frequency deviation.

**Figure 4:**
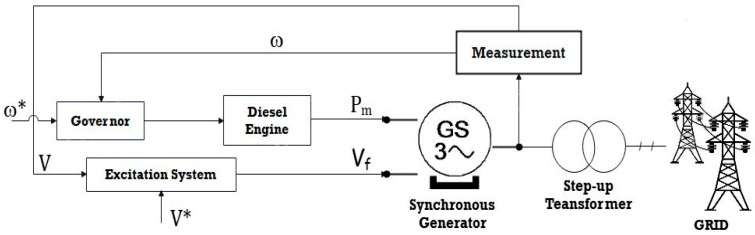
DG block diagram.

**Figure 5:**
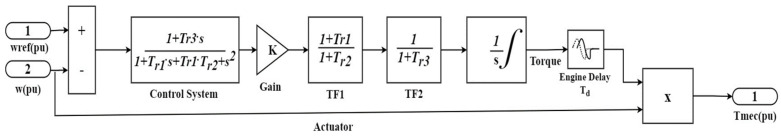
Block Diagrams for Governor and Diesel Generator Models.

Figure 4 and Figure 5 represent the DG and governor model block diagrams. The governor controls the rotational speed of the diesel engine to maintain a certain electrical frequency under various situations of electrical demand. The governor model is the Woodward diesel governor, which features a 5% frequency droop.

Based on the d-q rotor reference frame, a 3-phase synchronous machine represents a diesel generator. The following expression is used in the planning of the transfer function ($H_{df}$) of the diesel engine governor system:

(1) $$H_{d}=\frac{K_{g}(1+{sT}_{r3})}{\left(1+{sT}_{r1}+s^{2}T_{r1}T_{r2}\right)}$$

The equation shown below defines the actuator transfer function ($H_{a}$):

(2) $$H_{d}=\frac{K_{g}(1+{sT}_{a4})}{s(1+{sT}_{a5})(1+sT_{a6})}$$

where, *T_r_*_1_, *T_r_*_2_ and *T_r_*_3_ are the regulator time constants; *T_a_*_4_, *T_a_*_5_ and *T_a_*_6_ are the actuator time constants; *K_g_* is the regulator gain; and *T_d_* is engine time delay. Here, *T_r_*_1_, *T_r_*_2_ and *T_r_*_3_ are regarded as 0.01, 0.02, and 0.2, respectively; *T_a_*_4_, *T_a_*_5_ and *T_a_*_6_ are assumed to be equal to 0.25, 0.009, and 0.0384, respectively; *T_d_* is set at 0.024; mechanical power is assumed to have a starting value of 0.1361. The DG excitation system is realized by combining an exciter and an IEEE Type-1 voltage regulator of a synchronous machine.

### 2.2. PV Farm

**Figure 6:**
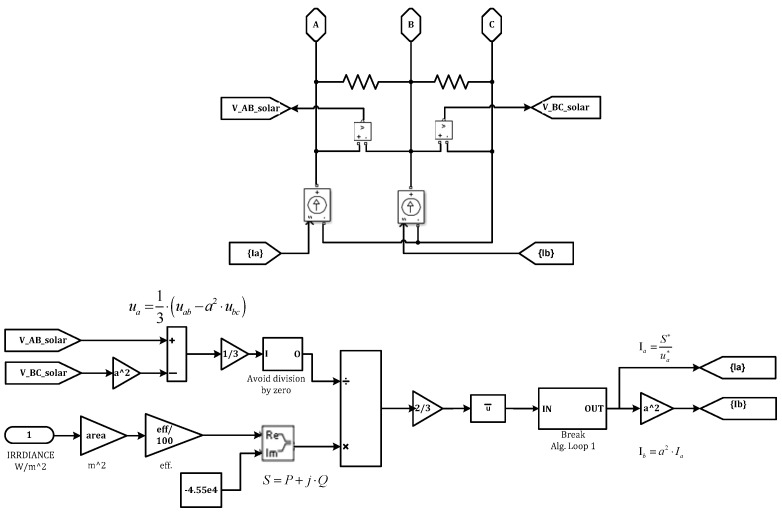
Block Diagrams for PV Generator.

The initial source of RES in this microgrid is a PV farm, which produces energy in accordance with three variables: the area it distributes electricity, the panel efficiency, and the irradiance data. Solar insolation is actuated in real-time using a simulation of irradiance. Additionally, 300 s of partial shadings are replicated for 12 h. Four variables are used in the PV subsystem calculation section to determine the current, which is subsequently inserted into the grid using AC sources, as shown in Figure 6. These are the phase-to-phase AC voltages, the solar radiation’s properties, the solar panels’ total useable area and their efficiency. The line voltage is converted towards the phase voltage based on the input values:

(3) $$U_{A}=\frac{1}{3}(U_{AB}-a^{2}U_{BC})$$

where, a is a complex Fortescue operator, U_A_ is a phase voltage in volt, and U_AB_ and U_BC_ are line-to-line voltages in volt.

By using Equation (4), the active power is determined:

(4) $$P={SR}_{i}*AREA*EFF$$

where *AREA* denotes the entire useable area of solar panels, *SRi* denotes the instantaneous amount of solar radiation, and *EFF* denotes the efficiency of the *PV* generator. Following the expression of the phase current:

(5) $$I_{A}=\frac{S^{*}}{U_{A}^{*}}$$

where *S* denotes the apparent power, and *U_A_* is a phase voltage.

A complex vector, also known as the modern complex Fortescue’s operator (a), is used to determine the value of the I_B_ for the examination of the steady-state performance of rotating machines.

(6) $$I_{B}=a^{2}I_{A}$$

### 2.3. Wind Farm

The next is a streamlined representation of a wind farm that generates wind-generated electricity. The wind farm begins to generate electricity as the wind speed reaches a particular point. Once the wind speed reaches the maximum permitted speed, the wind farm disconnects from the grid and remains disconnected until the wind decreases below the maximum. Table 2 shows the DG, PV, and wind parameters.

### 2.4. Vehicle to Grid

**Figure 7:**
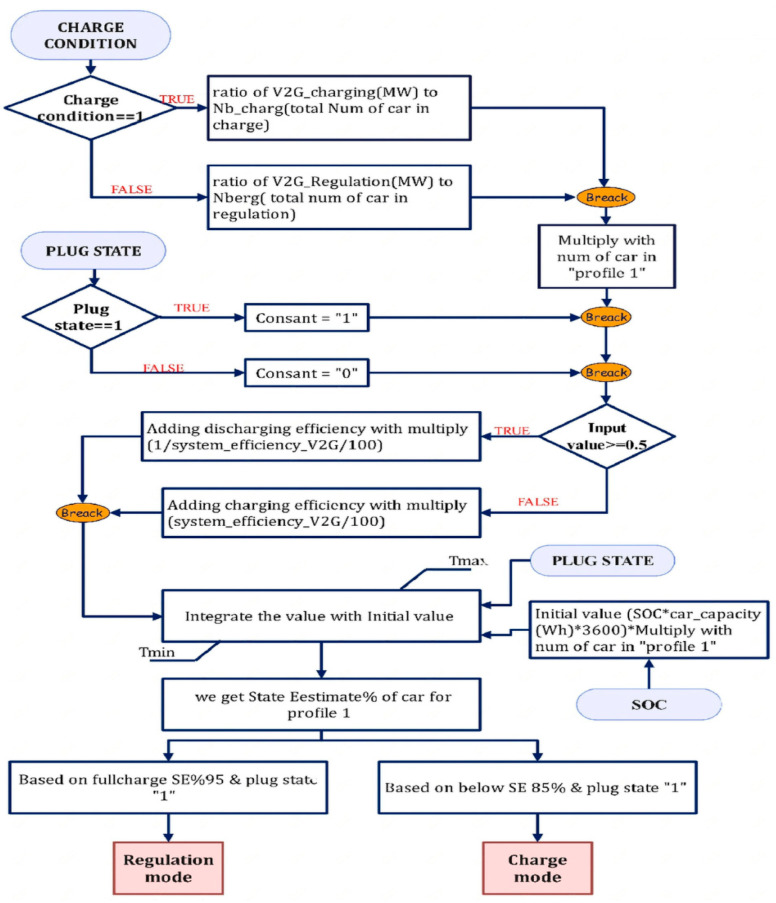
Flow Chart for Regulation and Charging Modes of V2G.

The V2G simulates a general collection of EVs. The mask may be adjusted to switch between the model’s five distinct profiles by modifying the plug-in and lookup tables of state of charge (SOC). The user may choose how many EVs will follow each sort of available port. The user may also select the power converter efficiency, rated volume, and rated power. The power output of each EV is 40 kW, and the V2G’s power rating is 16 MW consequently. To regulate the frequency of plug-in EVs (PHEVs), an aggregator is needed. The V2G aggregator monitors built into the grid’s network continually monitor the fleet of EVs. The whole profit depends on how many vehicles are V2G capable. Figure 7 shows the combined flow chart for the regulation and charging modes of V2G.

The energy that can be supplied to the fleet of EVs is included in the power. The operator can send signals or orders to the aggregator, which can then relay them to the fleet of EVs. As a result, it is possible to evaluate the PHEV fleet regulation capacity and choose the best bidding strategy. A specific PHEV’s involvement in regulating frequency via the bidirectional energy exchange will be decided and directed by the aggregator. The charge controller has a function when there is excess power in the grid or an over-frequency scenario, as well as when a significant industrial load is rejected or a source is brought back on. Two situations have been researched for charging the EVs while in charging mode. Plug-in first, then SOC second.

There are five groups made up of the whole EV fleet. Every group has a stochastic charging profile based on the amount of time needed to charge and dependent on the availability of charging stations. The SOC and plug-in time were used to profile EVs. The way to identify whether the EVs are in the charging state or the regulation condition is shown in Figure 6. Two limiters have also been implemented within the SOC controller architecture to restrict output variations brought on by plug-in state and SOC initialization. Figure 6 shows the Flow chart for the regulation and charging modes of V2G. The charger controller has the following features as described below:

I. The State Estimation (SE) has been set between 95% and 85%; outside that range, the charging process will stop to guarantee high-quality power output.
II. The EVs are in charging mode when SE is lower than 85% and in regulation mode when it is more than 95%.

**Figure 8:**
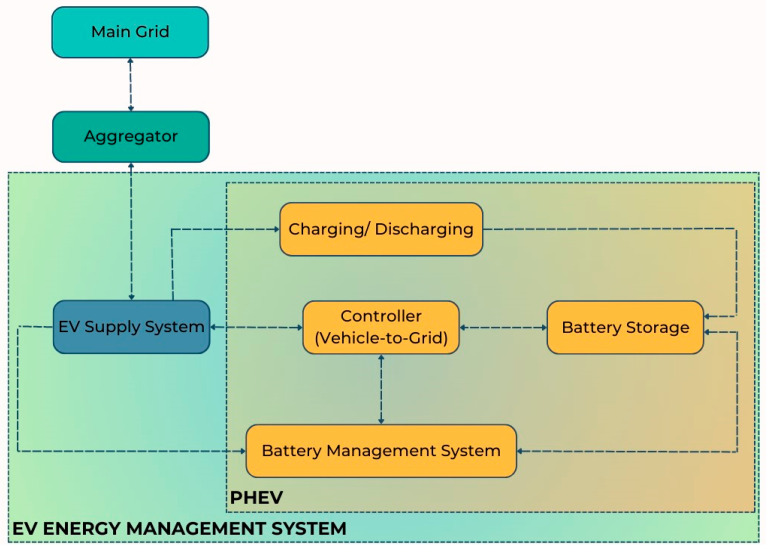
Framework of V2G operation.

Figure 8 summarises the V2G technology utilised for the study and illustrates the operating structure. To regulate the frequency of the PHEVs, an aggregator is needed. The V2G aggregator monitors built into the electricity grid’s network continually monitor the fleet of cars. The total number of vehicles with V2G capabilities determines the aggregate profile. The energy that can be supplied to the fleet of cars is included in the power. The operator can send signals or orders to the aggregator, which can then relay them to the fleet of cars. As a result, it is possible to evaluate the PHEV fleet’s regulation capacity and choose the best bidding strategy. The frequency regulation employing the bidirectional energy exchange will be decided and directed by the aggregator for a specific PHEV. The V2G controller uses the battery’s charge level and real-time frequency to make choices for the BMS. Charger/discharger block/sequence control is also provided by the V2G controller. Additionally, the battery’s healthy SOC is tracked by the BMS. Detailed statements of the controls utilised for the investigation are in [14].

### 2.5. Load

An asynchronous machine (ASM) and residential loads are utilized to model an industrial load effect on a system. These loads could be unique, like in ventilation systems. The load under consideration has a 20 MW overall capacity. The mechanical torque and rotor speed have a square relationship, which controls the ASM. At 3 h into the simulation, ASM is turned on. The ASM has a rating of 0.15 MW and 60 Hz frequency. Additionally, the networks on diesel generator bus, wind power generation bus, and load bus each have a 1 kW rating. Table 3 shows the Load parameters.

## 3. Results Discussion

The simulation of the model is for 24 h. The highest intensity of solar radiation happens in the middle of the day and follows a regular distribution range. Throughout the day, there are several peaks in the wind’s strength and brief bursts of minor amplitude. A typical pattern that closely approaches a pattern of residential load consumption is employed to simulate domestic load. The daytime consumption is minimal, and the evening hours are when it peaks, which progressively declines during the nighttime. During the day, the grid parameters would be affected by the following three circumstances:

I. The start of ASM was at the onset of the third hour.
II. At midday, some partial shadowing will be noticed, which impacts how much solar electricity is produced.
III. A wind farm trips every 22 h when the wind speed is higher than the highest allowed wind speed.

The study’s primary goal is to balance the system power to reduce changes in system characteristics like voltage and frequency and limit the amount of electricity imported from the utility network. The difference between the amount of energy produced by RES and ASMs and the amount of energy demanded by domestic, commercial, and EV load, as indicated below, determines the need for power balancing.

(7) $$P_{bal}=\left(P_{gen}+P_{w}+P_{pv}+P_{V2G}\right)-(P_{load}+P_{G2V})$$

*P_gen_*, *P_w_*, *P_pv_*, and *P_V_*_2*G*_ stand for the power generated by asynchronous machines, wind, solar and EVs discharge. Demand for residential, commercial, and PEV charging is represented by *P_load_* and *P_G_*_2*V*_. On the DG bus, whether to take power from or transfer power to the primary grid depends on whether the power balance is negative or positive.

The study focuses on reducing costs while simultaneously supplying consumers with high-quality electricity since the primary goal is to minimize energy conversation with the power grid and changes in system characteristics. This section provides a full analysis of the simulation results for modifications to the system’s characteristics, including voltage, current, active power, reactive power, and frequency for all units. This section also discusses the SOC outcomes for each of the five carpools. The results are analyzed to demonstrate the viability of the suggested design for the microgrid interfaced with V2G.

Some assumptions of the methodology are listed below:

- The simulation assumes that the microgrid runs continuously for the entire 24 h.
- The DG may have a linear frequency droop characteristic, according to the model. As a result, the frequency difference from the reference value directly affects how quickly the governor adjusts the speed.
- The model may assume that the sensors that measure the electrical frequency and the actuator that modifies the fuel supply to the engine react instantly and without any mistakes.
- The model assumes perfect transformers, inverters, and voltage regulators, as well as other lossless and ideal components. These components do not consider real-world losses like transformer or converter losses.
- Without considering realistic restrictions like charging/discharging rates and battery degradation, the V2G system is believed to have complete bidirectional power interchange with electric cars.

### 3.1. Diesel Generator Parameters

**Figure 9:**
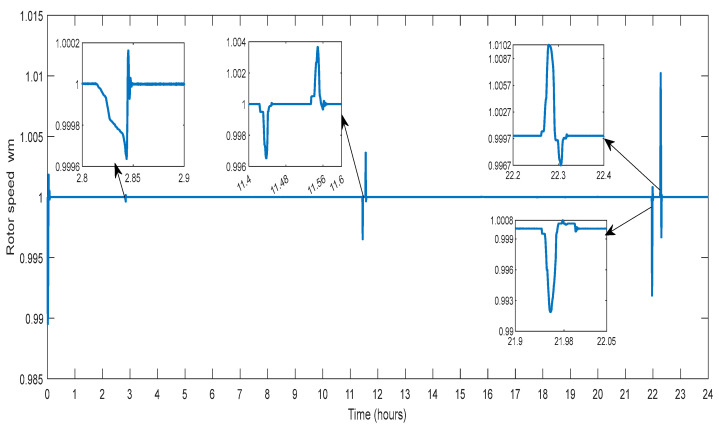
Rotor Speed of DG.

This section describes the outcomes of several factors for the typical DG. The governor rotor creates power in line with the rotor speed since it is directly connected to the generator shaft. The speed of the rotor is changed between hours 0.02 to 0.1, 2.8 to 2.9, 11.4 to 11.6, 21.9 to 22.05, and 22.2 to 22.4, to manage the power in the grid due to fluctuations in the operational situations caused by various profiles considered in the study. Figure 9 illustrates a DG that uses rotor speed variation to reduce the irregularity of the generation load balance.

**Figure 10:**
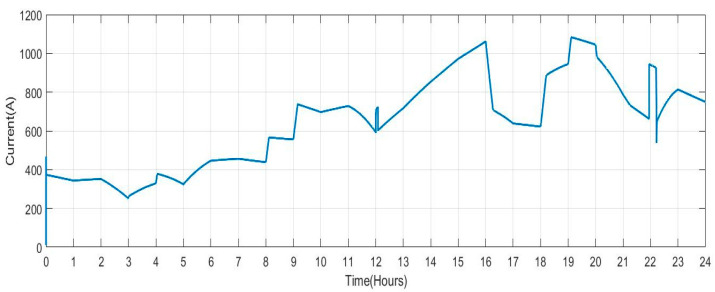
Current Measured on the Diesel Generator Bus.

Figure 10 displays the current that the DG supplies into the grid. The amount of current varies depending on the amount of electricity generated by RES and the amount of power exchanged with the V2G system. As a result, various spikes and a smaller current magnitude are seen in this figure.

**Figure 11:**
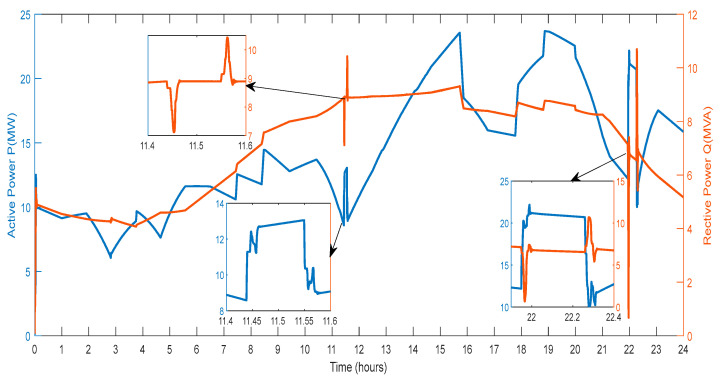
Active and Reactive Power of DG.

Figure 11 displays the active power and reactive power that the DG provides to the grid. The amount of active power varies depending on the power produced by wind farms, PV farms, and the power exchanged through the V2G system. Reactive power generation from RES and the conversation of reactive power with the V2G affect the amount of power in different ways. As a result, various spikes and a smaller active and reactive power magnitude are seen in Figure 11.

### 3.2. Wind Generator Parameters

**Figure 12:**
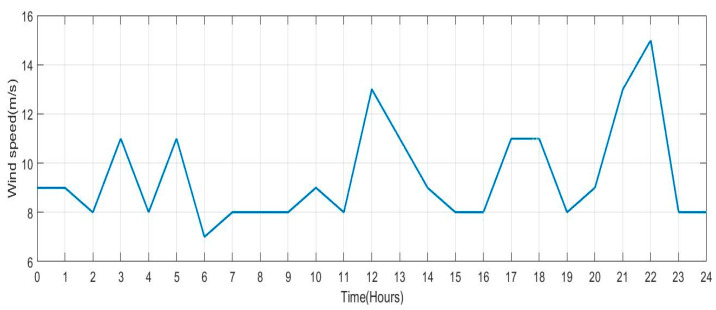
Wind Speed for 24 h.

This section describes the parameters recorded on the test system bus and changes in the wind turbine’s input wind speed. Deviations in wind speed over a 24-h period are regarded as inputs to the wind turbine to generate electricity, which is shown in Figure 12. From this graph, it can be seen that the minimal speed of wind is 7 m/s^2^ and that, depending on the time of day, wind speeds can range between 7 and 15 m/s^2^. This fluctuating wind speed will provide fluctuating power, simulating real-time fluctuations in wind power output.

**Figure 13:**
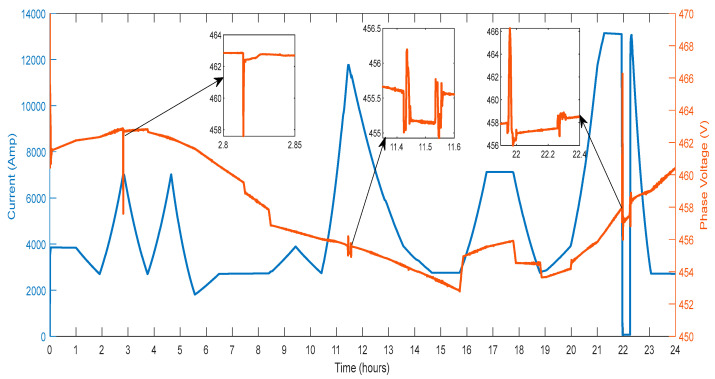
Wind Farm Phase Voltage and Current.

Also, the wind farm phase voltage and current variation are shown in Figure 13. According to changes in wind speed, the current strength increases or decreases. The amount of current the wind generator feeds into the electric grid grows as the wind speed increases. Additionally, as the wind speed diminishes, the utility grid receives less current from the wind generator. As a result, changes in wind speed affect the current that the wind generator produces.

**Figure 14:**
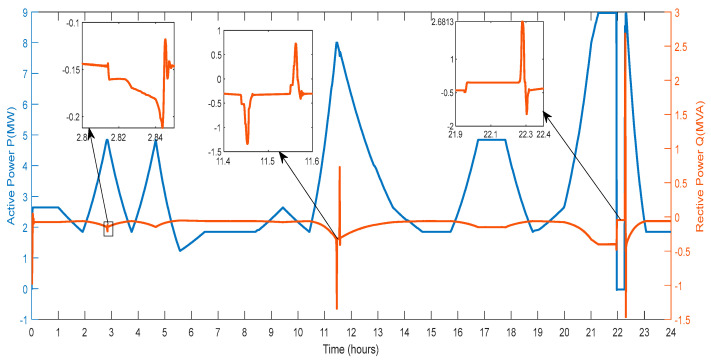
Active and Reactive Power of Wind Farm.

Figure 14 displays the active and reactive power of the wind farm provided to the grid. According to changes in wind speed, the power magnitude rises and falls. When the wind blows high, the wind generator will produce more energy to feed into the grid. The quantity of active power the wind generator can send to the utility grid decreases with low wind speed. As a result, changes in wind speed will affect the amount of active power the wind generator produces. Reactive power magnitude varies during three hours: 2.8 to 2.84, 11.4 to 11.6, and 21.9 to 22.4. This aids in controlling changes to the system parameters, particularly the microgrid voltage.

### 3.3. Solar Generator Parameters

**Figure 15:**
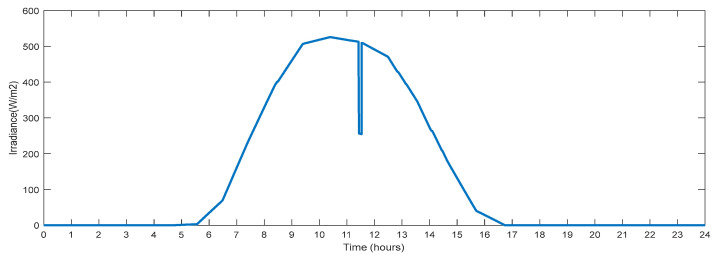
Solar Irradiance with Partial Shading.

This section describes the parameters recorded on the test system bus and changes in the input solar irradiation. Figure 15 shows changes in solar irradiation for one day that are taken for producing power from PV. The maximum irradiation is noted to be 520 (w/m^2^). A lowered value of the solar irradiation simulates partial shadowing for a duration of 300 s at the hour 11.5. This fluctuating solar irradiance will provide fluctuating power, simulating real-time fluctuations in solar power generation. At hour 5.7, solar irradiation begins to increase, and at hour 16.8, it reaches zero.

**Figure 16:**
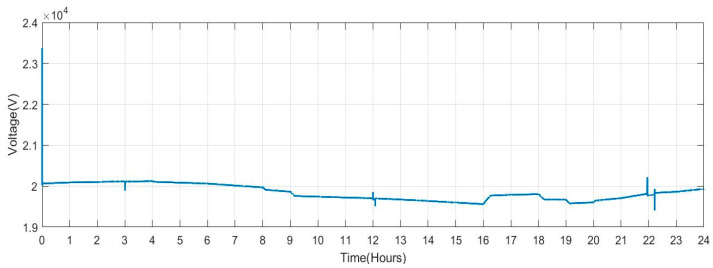
Voltage Measured at Solar PV Bus.

Figure 16 displays the voltage that was captured on bus B10. The voltage varies over nine time periods.

**Figure 17:**
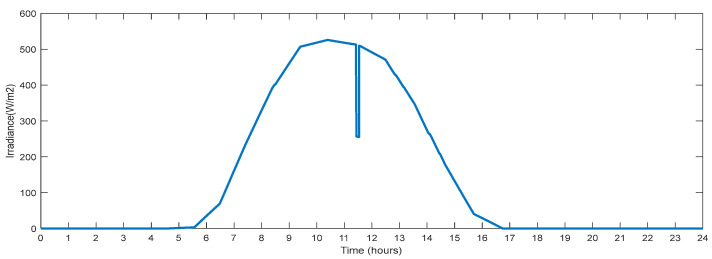
Current at PV Bus.

Figure 17 displays the current supplied to the grid by the PV generator. According to changes in irradiance, the current magnitude increases and decreases. The current provided to the grid by the PV generator will grow as the irradiance level rises. In addition, when the irradiance declines, so does the current so that the PV generator supplies to the grid. Thus, changes in irradiance will affect the current generated by the PV generator—additionally, the current drops after the solar PV panels are partially shaded.

**Figure 18:**
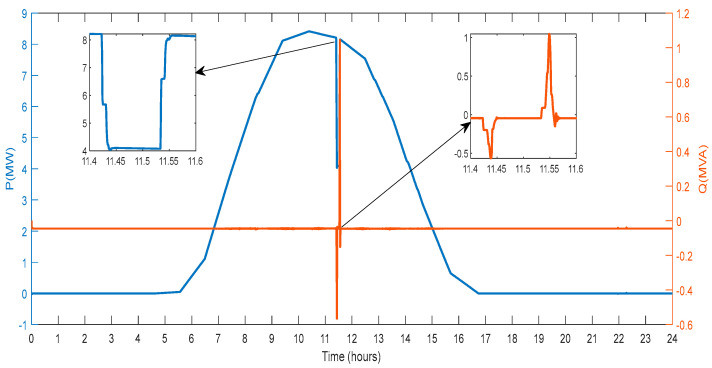
Active and Reactive Power at PV Bus.

Figure 18 displays the active and reactive power that the PV generator sent to the grid. Depending on the changes in irradiance, the amount of power generated increases and decreases. The amount of active power the PV generator generates and sends to the utility grid rises as the degree of irradiation increases. Additionally, as the irradiance drops, so decreases the power that the PV generator sends to the grid. Therefore, changes in irradiance will impact the amount of active power the solar PV generator can produce. After the solar PV plates were partially shaded, power output also decreased. In general, the power curve characteristics resemble those of irradiance. The microgrid and PV generator exchange reactive power between hours 11.4 to 11.6.

### 3.4. Consumer Load Parameters

**Figure 19:**
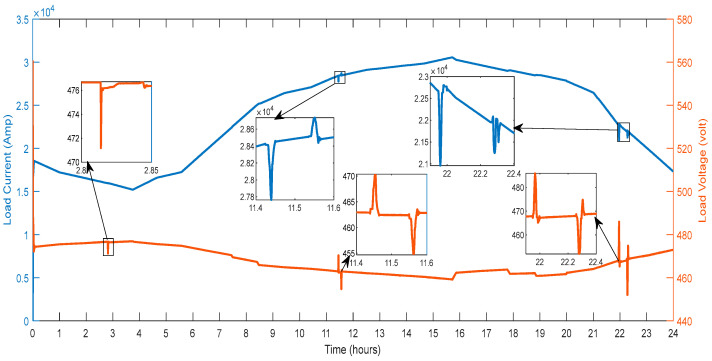
Voltage and Current of Residential Load.

The parameters connected with the consumer loads recorded on the system are described in detail in this section. Figure 19 shows the voltage and current recorded on the system’s bus. Between the periods of hours 21.8 and 22.4, a maximum voltage variation of 4.98% is seen in Figure 19. Between the hours of 2.8 and 2.85, there is a voltage sag of magnitude 0.413%. Transient components between hours 11.4 and 11.6 are detected to have magnitude amplitude variations of 0.39%. The transient components have amplitude variations of 3.73% and are seen between hours 22.2 and 22.40. Depending on changes in the load demand, the current’s magnitude rises and falls. The grid is used to draw a current in the 15,000 A to 30,000 A range, shown in Figure 19.

Additionally, as load demand fluctuates, local variances are seen. Hours 2.8 to 2.9, 11.4 to 11.6 and 21.8 to 22.4 show abrupt fluctuations in the demand of load. By employing the current balancing with the V2G, variations in load demand are reduced.

**Figure 20:**
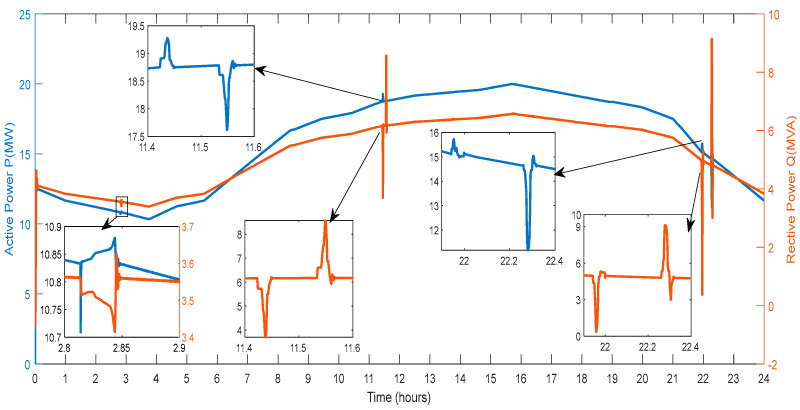
Active and Reactive Power of Residential Load.

Figure 20 shows the active and reactive power that the load draws from the microgrid. The load draws between 10 MW and 20 MW of active power from the microgrid, shown in Figure 20. Hours 2.8 to 2.9, 11.4 to 11.6 and 21.8 to 22.4 show abrupt fluctuations in the demand of load. The current balance employing the V2G has reduced deviations in the load demand. There are abrupt fluctuations in the demand for reactive power load at hours 11.4 to 11.6 and 21.8 to 22.4.

### 3.5. Industrial Load Parameters

**Figure 21:**
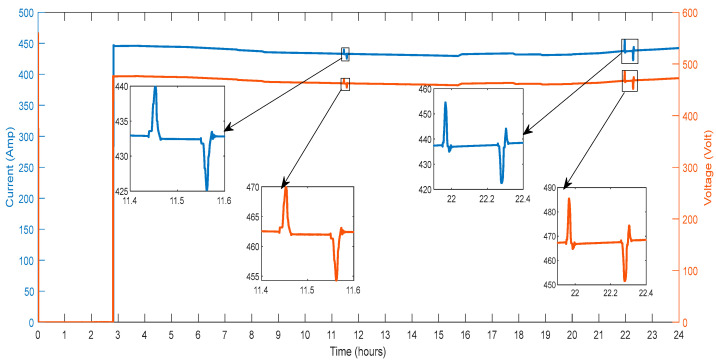
Voltage and Current of Asynchronous Machine.

This section describes the outcomes of parameters related to the ASM verified on bus B9 of the microgrid. Figure 21 depicts the voltage and current on the test bus B9. An asynchronous machine load of 0.15 MVA is turned on every three hours. It is perceived that on the ASM, the large value of inrush current, which is equal to 452 A received at the instant of switching. More deviations in the current are also detected at hours 11.4 to 11.6 and 21.8 to 22.4.

**Figure 22:**
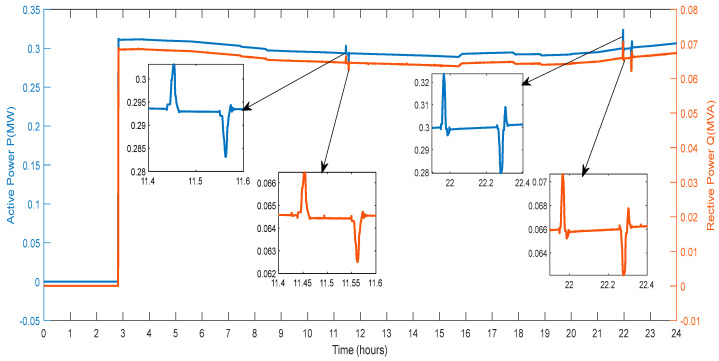
Active and Reactive Power of Asynchronous Machine.

Figure 22 displays the ASM’s active and reactive power draw from the grid. It is perceived that a large value of active power is received during switching on the ASM. Consequently, the ASM is consumed continuous active power of 0.32 MW. Additional deviations in the active power are detected at the hour 11.4 to 11.6 and 21.8 to 22.4. Figure 22 displays the ASM’s reactive power draw from the grid. It is perceived that a large value of active power is received during switching on the ASM. Consequently, the ASM is consumed continuous reactive power of 0.07 MVA. Additional deviations in the reactive power are detected at 11.4 to 11.6 and 21.8 to 22.4.

### 3.6. V2G Regulation Parameters

**Figure 23:**
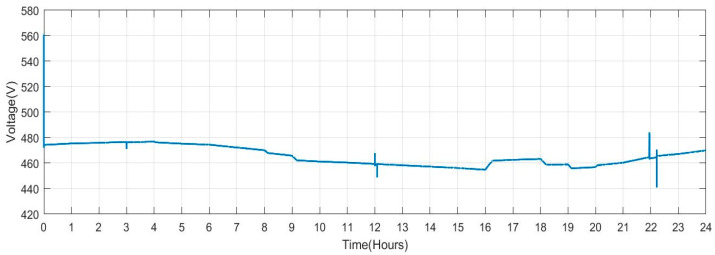
V2G Voltage at Bus B8.

This section describes the outcomes of the parameters related to the regulation of V2G on bus B8 as shown in Figure 23.

**Figure 24:**
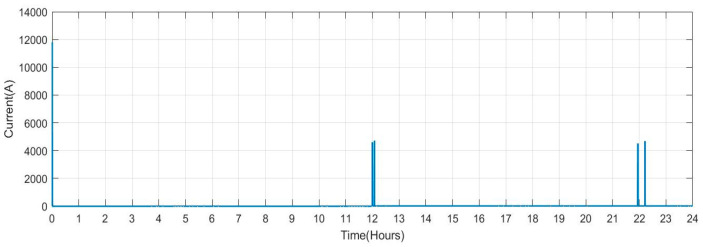
V2G Current at Bus B8.

Figure 24 shows the current received for regulations of the deviation by the V2G. It is seen that the current is received at the hour 11.4 to 11.6 and 21.8 to 22.4, with high magnitude.

**Figure 25:**
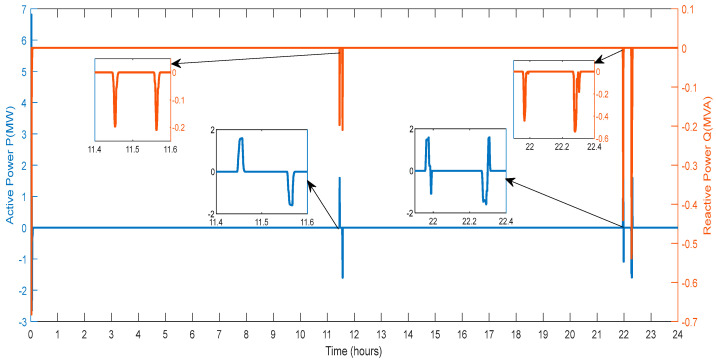
Active and Reactive Power of V2G Regulation.

Figure 25 shows the exchange of active and reactive power for the regulation of the deviations by the V2G. The active and reactive power is swapped at the hour 11.4 to 11.6 and 21.8 to 22.4, with high-magnitude peaks, where the first peak shows that the V2G draws the active power and another magnitude shows that the active power is delivered to the V2G to diminish the deviations.

### 3.7. V2G Charging Parameters

The specifications for V2G for the charging of vehicle batteries are described in this section. Seven-time periods of voltage variations are present. The five scenarios are taken for the study, where a probable duration for the car’s charging is also stated.

Scenario 1: Persons who commute to work have the option of charging their vehicles there (140 vehicles altogether).

Scenario 2: Persons that commute to work and charge their vehicles there might expect a longer journey time. (100 vehicles altogether).

Scenario 3: Persons who commute to work do not charge their vehicles there (40 vehicles altogether).

Scenario 4: Persons who remain at home (80 vehicles altogether).

Scenario 5: Persons doing the night shift (40 vehicles altogether).

**Figure 26:**
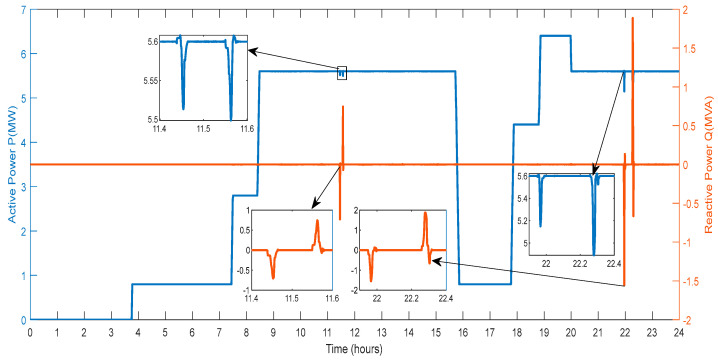
Active and Reactive Power of V2G Charging.

Figure 26 displays the active and reactive power calculated during charging V2G vehicles. The power drained from the grid is marked as “negative”. As a result, batteries use the grid for the four time periods that were previously mentioned. For charging batteries, vehicles consumed reactive power from the grid during hours 11.4 to 11.6 and 21.8 to 22.4. When the batteries are connected to charging, the reactive power is given by the batteries, and when the batteries are disconnected, the reactive power is obtained from the grid.

In Table 4, the suggested system’s sensitivity analysis is displayed. When measuring frequency and voltage under various V2G vehicle scenarios, it has been shown that the inaccuracy is below 5%. Because of this, the suggested model for V2G and microgrid is not overly sensitive to changes in operational situations and successfully runs in various operating situations.

**Figure 27:**
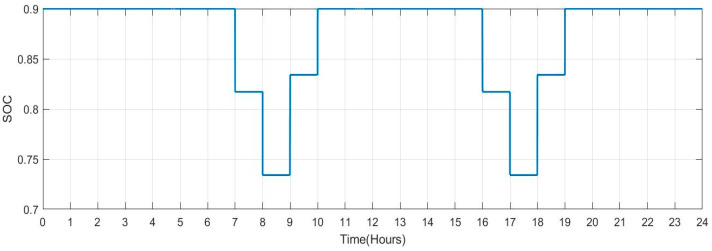
SOC Data of Scenario 1: [0.9 0.9 0.9 0.9 0.9 0.9 0.9 1 1 0.834 0.9 0.9 0.9 0.9 0.9 0.9 1 1 0.834 0.9 0.9 0.9 0.9 0.9].

**Figure 28:**
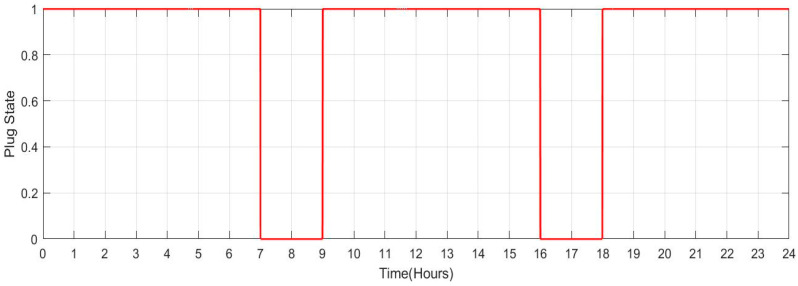
Plug State of Car Scenario 1: [1 1 1 1 1 1 1 0 0 1 1 1 1 1 1 1 0 0 1 1 1 1 1 1].

Table 5 shows the SOC value and plug state for each car profile. Figure 27 and Figure 28 depict Scenario 1: this scenario indicates persons commuting to work with a facility charging their vehicles there (no. of vehicles:140). As soon as the car is unplugged, the max SOC set to 0.9 changes to 1 and has a lowered value of 0.817 with respect to time (hour). Its decreasing rate is 8.3%. However, when the car is plugged back in, its increasing rate is 10%.

**Figure 29:**
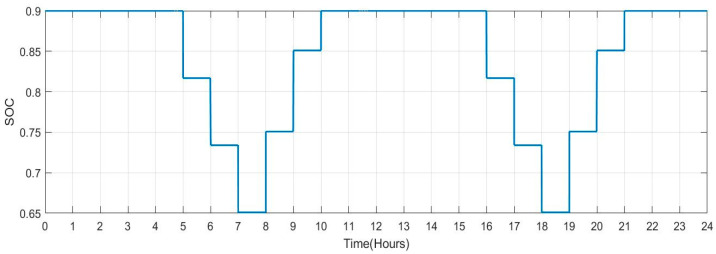
SOC Data of Scenario 2: [0.9 0.9 0.9 0.9 0.9 0.817 0.734 0.651 0.751 0.851 0.9 0.9 0.9 0.9 0.9 0.9 0.817 0.734 0.651 0.751 0.851 0.9 0.9 0.9].

**Figure 30:**
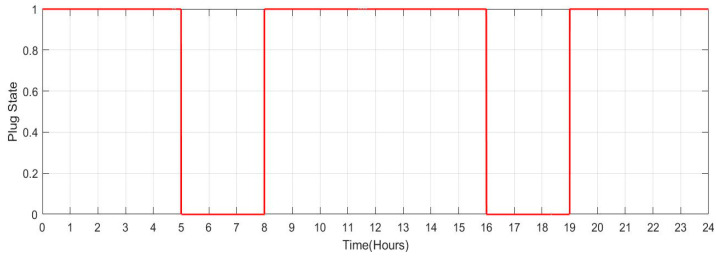
Plug State of car Scenario 2: [1 1 1 1 1 0 0 0 1 1 1 1 1 1 1 1 0 0 0 1 1 1 1 1].

Figure 29 shows the SOC of batteries for Scenario 2: individuals who commute to work with a facility for charging their vehicles but with a lengthier journey (no. of vehicles: 100) during a one-day period. When people make a long trip from their house to their place of work in the morning between the hours of 5:00 to 8:00, it is seen that the SOC of the batteries is lower. Similarly, the SOC also drops when individuals leave work in the late afternoon or evening between 16:00 and 19:00. When batteries are charged at work and reach highs of more than 90. SOC rises during this time. Figure 30 shows the plug state of car Scenario 2.

**Figure 31:**
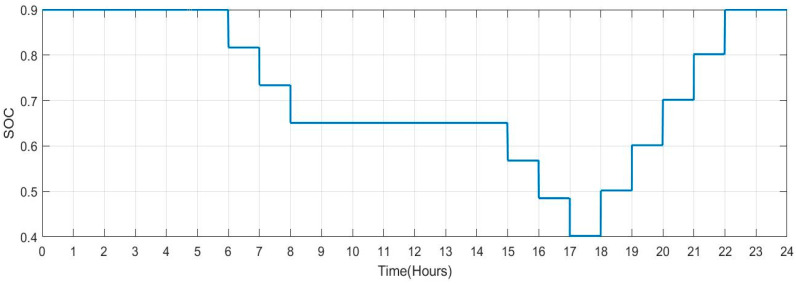
SOC Data of Scenario 3: [0.9 0.9 0.9 0.9 0.9 0.9 0.817 0.734 0.651 0.651 0.651 0.651 0.651 0.651 0.651 0.568 0.485 0.402 0.502 0.602 0.702 0.802 0.9 0.9].

**Figure 32:**
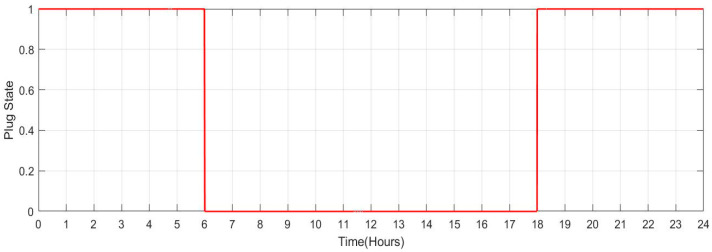
Plug State of car Scenario 3: [1 1 1 1 1 1 0 0 0 0 0 0 0 0 0 0 0 0 1 1 1 1 1 1].

Figure 31 shows the SOC of batteries for Scenario 3 over a 24-h period when people arrive at work but are unable to charge their vehicles (number of vehicles: 10). When travelling from home to work in the morning and returning home from hours 15:00 to 18:00 in the evening, the SOC of the batteries drops in this scenario by 8.3%. Figure 32 shows the plug state of car Scenario 3.

**Figure 33:**
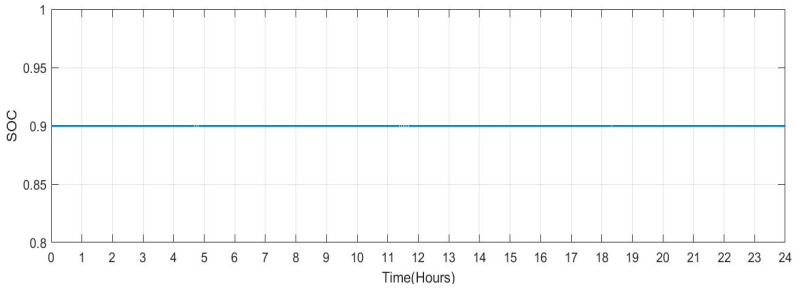
SOC Data of Scenario 4: [0.9 0.9 0.9 0.9 0.9 0.9 0.9 0.9 0.9 0.9 0.9 0.9 0.9 0.9 0.9 0.9 0.9 0.9 0.9 0.9 0.9 0.9 0.9 0.9].

**Figure 34:**
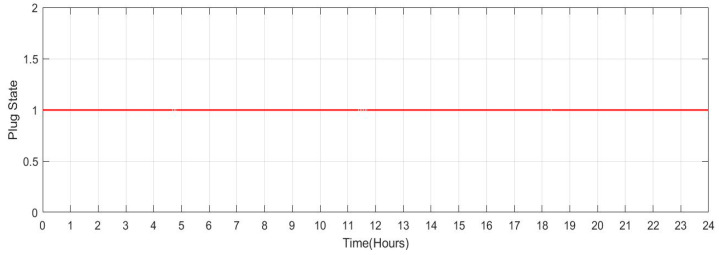
Plug State of car Scenario 4: [1 1 1 1 1 1 1 1 1 1 1 1 1 1 1 1 1 1 1 1 1 1 1 1].

Figure 33 shows the SOC of the batteries for Scenario 4 throughout a 24-h period when the individuals remain at home (number of vehicles: 20). It has been noted that the batteries’ SOC has been nearly consistent throughout. However, when cars are operated in grid regulation mode, minor variances are seen. Figure 34 shows the plug state of car Scenario 4.

**Figure 35:**
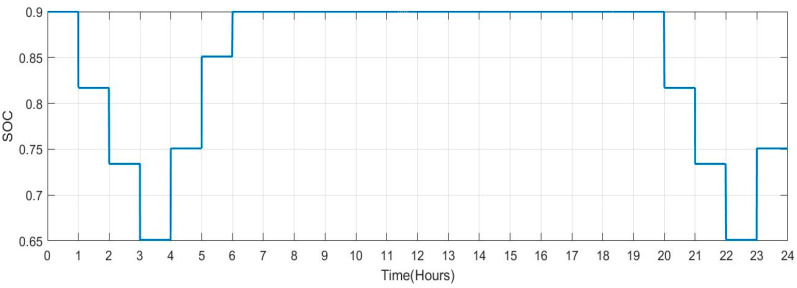
SOC Data of car Scenario 5: [0.9 0.817 0.734 0.651 0.751 0.851 0.9 0.9 0.9 0.9 0.9 0.9 0.9 0.9 0.9 0.9 0.9 0.9 0.9 0.9 0.817 0.734 0.651 0.751].

**Figure 36:**
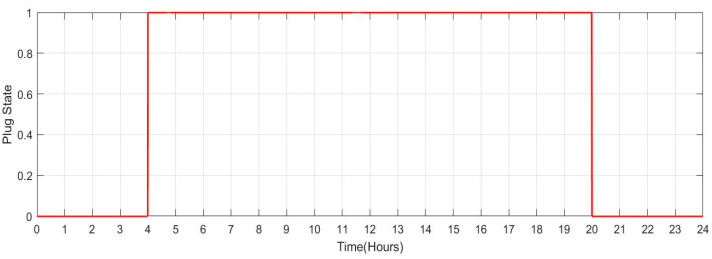
Plug State of car Scenario 5: [0 0 0 0 1 1 1 1 1 1 1 1 1 1 1 1 1 1 1 1 0 0 0 0].

Figure 35 shows the SOC of the batteries for Scenario 5 during a 24-h period while individuals are working the night shift (10 total vehicles). When people commute from home to work during the night shift, between the hours of 20 and 4 the following day, the SOC of the batteries decreases in this situation. During daylight hours, when individuals are at home, the likelihood of SOC rises. Because the batteries are not charged at work, the SOC is maintained at low levels throughout the night. Figure 36 shows the plug state of car Scenario 5.

**Figure 37:**
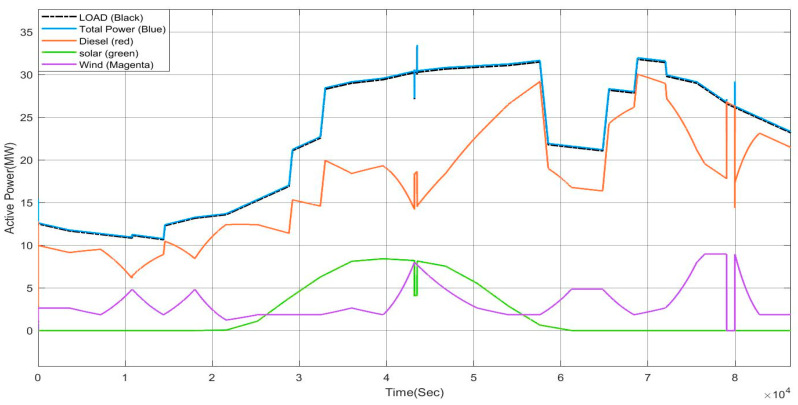
Active Power of V2G.

Figure 37 depicts the active power of the generating sources and the residential load, industrial load, and V2G load. Throughout the 24 h the power will be balanced, as shown in Figure 37. Here we have the previously discussed scenario of partial shading at noon when generated power is suddenly reduced so that a diesel generator will provide power. When wind speed goes above the speed limit, that wind generator will be cut-off at the hour 22:00, then the diesel generator offers total power to the load.

The limitations of the proposed methodology are listed below:

- The accuracy of the model’s representation of real-world behaviour may not have been validated against actual microgrid data or field measurements.
- The V2G model does not provide specific details about different types of EVs (e.g., plug-in hybrid electric vehicles, battery electric vehicles), each of which might have unique charging and discharging characteristics.
- The model does not consider environmental factors, such as temperature and weather, which can influence charging and discharging performance.

## 4. Conclusions

Although V2G operation may shorten the battery life of vehicles, it is anticipated to be cost-effective for grid operators and car owners. Ancillary services like voltage and frequency control and spinning reserves can be made possible by the V2G concept. To satisfy the demand and control the parameters of the microgrid, a V2G system interfaced with a microgrid has been proposed for a 24-h cycle. A DG, RESs, a V2G system, and the loads are the four parts that make up a microgrid. A microgrid is built to reflect a community of a thousand homes daily with low demand in the spring or fall. To realize 1:5 ratios between EVs and homes, which may be a potential situation in the near future, a total of 400 EVs have been demonstrated as basic models. The suggested microgrid and V2G system successfully ran, satisfying the load demand. By feeding energy from the EV batteries to the grid, V2G has been used to control changes in the microgrid parameters. All the data have been analyzed, including voltage, active and reactive power, current, fluctuations in wind speed and irradiance, and battery SOC, and it has been concluded that the proposed V2G and microgrid architecture successfully controls all of these variables.

The microgrid and V2G characteristics have been controlled through trial and error. It is observed that the V2G operates effectively in various operating scenarios, and the measurement error of frequency and voltage for various V2G vehicle scenarios is below 5%, showing that the suggested V2G model and microgrid are not particularly sensitive to the changes in operating conditions. To efficiently manage the loads linked to the grid, optimization techniques may be utilized in the future to enhance the functionality and efficacy of the energy resources.

Future increases in EV adoption would result in a greater overall strain on the grid. This illustrates a future scenario where the system operator could invest in a well-organized aggregation of EVs rather than new generating units. To encourage owners of EVs to actively contribute to grid stability and provide an additional source of revenue, new incentives would be required.

## Figures and Tables

**Table 1:** Current zero-emission light-duty vehicle (ZEV) incentives in selected countries [8].

		Canada	China	European Union	India	Japan	USA
Incentives vehicle	Fiscal incentives	✓	✓	✓	✓	✓	✓
Regulations charger	Hardware standards	✓	✓	✓	✓	✓	✓
	Building regulations	✓	✓	✓	✓	---	✓
Incentives charger	Fiscal incentives	✓	✓	✓	✓	✓	✓

**Table 2:** Parameters of DG, PV, and Wind.

Technical Parameter	Value
Diesel Generator	40 MW
Nominal Frequency	60 Hz
PV Farm Power	8 MW
PV Farm Efficiency	20%
PV Farm Area	8000 m^2^
Wind Farm Area	9 MW
Nominal Wind Speed	13.5 m/S^2^
Maximum Wind Speed	15 m/S^2^

**Table 3:** Load Parameters.

Technical Parameter	Value
V2G Rated Power	40 MW (per Car)
Rated Capacity	85 kWh (per Car)
V2G Efficiency	90%
Total Cars	400
Domestic Load	20 MW
Power Factor	0.95
Time-Step	60 min
Asynchronous Machine Load	0.15 MVA

**Table 4:** Sensitivity Analysis under various V2G scenarios.

	Scenario	Number of EVs				
		Profile 1	Profile 2	Profile 3	Profile 4	Profile 5
	1	140	120	100	80	120
	2	100	100	80	120	120
	3	40	60	40	60	40
	4	80	80	100	60	80
	5	40	40	80	80	40
Change in Parameter	Voltage	3.87%	3.47%	3.53%	3.69%	3.49%
	Frequency	0252%	0.232%	0.247%	0.260%	0.234%
Error pertaining to Scenario 1	Voltage	0.00%	3.72%	3.67%	3.81%	3.20%
	Frequency	0.00%	2.90%	3.15%	3.37%	2.35%

**Table 5:** SOC value and plug state for each car profile.

Car Profiles	SOC	Plug State
1	[0.9 0.9 0.9 0.9 0.9 0.9 0.9 1 1 0.834 0.9 0.9 0.9 0.9 0.9 0.9 1 1 0.834 0.9 0.9 0.9 0.9 0.9]	[1 1 1 1 1 1 1 0 0 1 1 1 1 1 1 1 0 0 1 1 1 1 1 1]
2	[0.9 0.9 0.9 0.9 0.9 0.817 0.734 0.651 0.751 0.851 0.9 0.9 0.9 0.9 0.9 0.9 0.817 0.734 0.651 0.751 0.851 0.9 0.9 0.9]	[1 1 1 1 1 0 0 0 1 1 1 1 1 1 1 1 0 0 0 1 1 1 1 1]
3	[0.9 0.9 0.9 0.9 0.9 0.9 0.817 0.734 0.651 0.651 0.651 0.651 0.651 0.651 0.651 0.568 0.485 0.402 0.502 0.602 0.702 0.802 0.9 0.9]	[1 1 1 1 1 1 0 0 0 0 0 0 0 0 0 0 0 0 1 1 1 1 1 1]
4	[0.9 0.9 0.9 0.9 0.9 0.9 0.9 0.9 0.9 0.9 0.9 0.9 0.9 0.9 0.9 0.9 0.9 0.9 0.9 0.9 0.9 0.9 0.9 0.9]	[1 1 1 1 1 1 1 1 1 1 1 1 1 1 1 1 1 1 1 1 1 1 1 1]
5	[0.9 0.817 0.734 0.651 0.751 0.851 0.9 0.9 0.9 0.9 0.9 0.9 0.9 0.9 0.9 0.9 0.9 0.9 0.9 0.9 0.817 0.734 0.651 0.751]	[0 0 0 0 1 1 1 1 1 1 1 1 1 1 1 1 1 1 1 1 0 0 0 0]

## Data Availability

The data presented in this study are available on request from the corresponding author.
